# Acute regional improvement of myocardial function after interventional transfemoral aortic valve replacement in aortic stenosis: A speckle tracking echocardiography study

**DOI:** 10.1186/1476-7120-10-15

**Published:** 2012-03-26

**Authors:** Sebastian Schattke, Gerd Baldenhofer, Ines Prauka, Kun Zhang, Michael Laule, Verena Stangl, Wasiem Sanad, Sebastian Spethmann, Adrian C Borges, Gert Baumann, Karl Stangl, Fabian Knebel

**Affiliations:** 1 Charité - Universitätsmedizin Berlin, Medizinische Klinik m.S. Kardiologie und Angiologie, Charité Campus Mitte, Charitéplatz 1, 10117 Berlin, Germany; 2 Klinik für Innere Medizin I, Kardiologie und Diabetologie, Helios Klinikum Emil von Behring, Berlin, Walterhöferstraße 11, 14165 Berlin, Germany; 3 Klinik am See, Abteilung für Kardiologie, Seebad 84, 15562 Rüdersdorf, Germany

## Abstract

**Background:**

Transcatheter aortic valve implantation (TAVI) is a promising therapy for patients with severe aortic stenosis (AS) and high perioperative risk. New echocardiographic methods, including 2D Strain analysis, allow the more accurate measurement of left ventricular (LV) systolic function. The goal of this study was to describe the course of LV reverse remodelling immediately after TAVI in a broad spectrum of patients with symptomatic severe aortic valve stenosis.

**Methods:**

Thirty consecutive patients with symptomatic aortic valve stenosis and preserved LVEF underwent transfemoral aortic valve implantation. We performed echocardiography at baseline and one week after TAVI. Echocardiography included standard 2D and Doppler analysis of global systolic and diastolic function as well as 2D Strain measurements of longitudinal, radial and circumferential LV motion and Tissue Doppler echocardiography.

**Results:**

The baseline biplane LVEF was 57 ± 8.2%, the mean pressure gradient was 46.8 ± 17.2 mmHg and the mean valve area was 0.73 ± 0.27 cm^2^. The average global longitudinal 2D strain of the left ventricle improved significantly from -15.1 (± 3.0) to -17.5 (± 2.4) % (p < .001). This was reflected mainly in improvement in the basal and medial segments while strain in the apex did not change significantly [-11.6 (± 5.2) % to -15.1 (± 5.5) % (p < .001), -13.9 (± 5.1) % to -16.8 (± 5.6) % (p < .001) and -19.2 (± 7.0) % to -20.0 (± 7.2) % (p = .481) respectively]. While circumferential strain [-18.1 (± 5.1) % vs. -18.9 (± 4.2) %, p = .607], radial strain [36.5 (± 13.7) % vs. 39.7 (± 17.2) %, p = .458] and the LVEF remained unchanged after one week [57.0 (± 8.2) % vs. 59.1 (± 8.1) %, p = .116].

**Conclusion:**

There is an acute improvement of myocardial longitudinal systolic function of the basal and medial segments measured by 2D Strain analysis immediately after TAVI. The radial, circumferential strain and LVEF does not change significantly in all patients acutely after TAVI. These data suggest that sensitive new echo methods can reliably detect early regional changes of myocardial function after TAVI before benefits in LVEF are detectable.

## Introduction

The prevalence of calcific aortic stenosis is increasing in ageing societies. Thus the number of patients with associated co-morbidities and high operative risk is increasing [1,2]. Transcatheter aortic valve implantation (TAVI) with either self-expandable or balloon-expandable valved stents is a promising therapy for aortic stenosis (AS) in these high risk patients [3,4]. Several recently published surveys and studies have shown the feasibility and safety of the new invasive method in short and mid-term follow up period [5-9].

Long standing AS leads to elevated left ventricular (LV) pressure and as a consequence develop LV hypertrophy and increased myocardial fibrosis. The LV ejection fraction (EF) remains often preserved until late stages of disease but it represents only a part of the entire myocardial contraction. It has been reported that despite of normal EF the LV myocardial long axis excursion measured by M-mode echocardiography is reduced in patients with severe AS [10]. Newer echocardiographic methods like tissue Doppler imaging and 2D speckle tracking analysis allow assessment of myocardial deformation as new sensitive marker for regional and global LV systolic function [11-16].

Recently published studies have demonstrated an improvement in LV systolic function assessed by tissue Doppler and speckle tracking strain imaging in patients with severe AS and preserved EF after conventional surgical aortic valve replacement in a mid-term and long term follow up [17-21].

One study has compared the acute left ventricular reverse remodelling after percutaneous and surgical aortic valve replacement but only with conventional echo parameters [22].

The aim of the present study was to detect acute regional changes of LV myocardial deformation parameters assessed by 2D speckle tracking strain analysis as a reflection of LV reverse remodelling immediately after TAVI in very old and co-morbid patients with symptomatic severe aortic valve stenosis who were not able to undergo surgical valve replacement.

## Methods

### Study population

Thirty consecutive patients with symptomatic aortic valve stenosis and preserved EF were included in this prospective study. The operative risk of all patients was calculated according to the logistic European System for Cardiac Operative Risk Evaluation score [23,24]. During screening period all patients underwent physical examination, electrocardiography, transthoracic and transesophageal Doppler echocardiography, coronary angiography and pelvic angiography. Patients with significant coronary artery disease were fully revascularized by percutaneous coronary intervention prior to TAVI. The inclusion criteria for TAVI were either compassionate use in some cases or the criteria described previously (5). Written informed consent was obtained from each patient.

### Echocardiography

Standard transthoracic echocardiography including Doppler analysis was performed according to the guidelines of the American Society of Echocardiography [23] on Vivid 7 Dimension (GE Vingmed, Horton, Norway, M4S 1.5-4.0 MHz transducer). Patients were imaged in the left lateral decubitus position. LV volumes were measured and EF was calculated according to the modified Simpson's rule using the apical 4- and 2-chamber views.

Mean and maximum aortic valve pressure gradient were estimated by the modified Bernoulli equation and the flow velocity time integral over the ejection period on continuous wave Doppler recordings. The aortic valve area was determined by the continuity equation following the recommendations of the American Society of Echocardiography.

### 2D speckle-tracking strain analysis

For assessment of radial, circumferential and longitudinal speckle tracking strain and strain rate standard 2D ultrasound images at parasternal mid-ventricular short axis view (at the level of papillary muscle) and from the apical long axis, two chamber- and four-chamber views with a frame rate between 40 and 80 frames per second (fps) were recorded and stored digitally for offline analysis (EchoPac PC, GE Vingmed, Horton Norway) as previously described [11-13].

After manually tracing of endocardial borders the software automatically traced the region of interest including the entire myocardial wall. In this process the left ventricle was divided into six segments. To optimize the tracking the region of interest width was adjusted, if needed. For each segment the quality of speckle tracking was analyzed automatically. Segments with poor tracking were excluded for further measurements.

Peak systolic longitudinal strain and strain rate of the apical two-, four-chamber and long axis view were calculated averaging the peak systolic strain- and strain rate values of the six segments of the corresponding views. Finally, the global longitudinal peak systolic strain and strain rate of the left ventricle were generated averaging peak systolic values of the three apical views. Peak systolic radial and circumferential strain and strain rate were calculated averaging the values of the six LV segments from parasternal mid-ventricular short axis view. Since the papillary muscles yield good anatomic landmarks in order to improve reproducibility for follow-up examinations.

### Inter- and intra-observer variability analysis

Three echocardiographers, blinded to clinical data, independently measured strain and strain rate of 10 randomized patients (five cases prior TAVI and five patients post TAVI) for interobserver variability analysis. One experienced observer calculated strain and strain rate twice on two consecutive days for analysis of intra-observer variability.

### Statistics

All results are expressed as mean value ± standard deviation (SD). The Mann-Whitney nonparametric test was used to compare echocardiographic data from baseline and follow up values. Differences were considered statistically significant if the P value was less than 0.05. Interclass Correlation Coefficient by Kolmogorov-Smirnov was used to calculate the intra-and inter-observer variability. Statistics were calculated by software (SPSS, Version 18.0, SPSS Inc, Chicago, Ill).

## Results

Thirty patients with symptomatic aortic stenosis were included in this monocentric study. The baseline characteristics are presented in Table 1. 15 patients received Core Valve 29 mm, in 12 patients Core Valve 26 mm were implanted, two patients received Sapien 26 mm and one patient Sapien 23 mm. All patients were hemodynamically stable. No patient died within one week after TAVI.

**Table 1:** Baseline characteristics

Age (years)	80.9 (67 - 92)
Sex (female)	19 (63%)

EuroSCORE (%)	18.3 ± 11.9

Body mass index (kg/m^2^)	26.2 ± 5.5

Diabetes mellitus	9 (30 %)

Atrial fibrillation	4 (13%)

Permanent pacemaker	5 (17%)

Arterial hypertension	28 (93%)

Coronary artery disease	18 (60%)

COPD	9 (30%)

NYHA III or IV	15 (50%)

NYHA II	15 (50%)

History of syncope	4 (13%)

Combined mild AI	18 (60%)

Combined moderate AI	4 (13%)

Mild MI	13 (43%)

Moderate MI	11 (37%)

Data are expressed as mean ± SD or as number (percentage or range). EuroSCORE, European System for Cardiac Operative Risk Evaluation; COPD, chronic obstructive pulmonary disease; AI, aortic valve insufficiency; MI, mitral valve insufficiency

Four patients had atrial fibrillation at baseline. After TAVI, five patients were examined in atrial fibrillation with normal heart rate. Five patients had permanent pacemakers implanted prior to TAVI. Two patients needed a de novo permanent pacemaker implantation after TAVI due to AV conduction abnormalities.

### Standard echocardiograhic measures

The changes of left ventricular volumes, dimensions and function as well as Doppler values are given in Table 2. Irrespective of significant pressure load reduction LV volumes and LVEF remained unchanged. There were no significant differences in heart rate between baseline and follow up. According to LV diastolic function the transmitral early diastolic flow velocities (E) increased significantly as well as the myocardial early diastolic velocities (E') at the basal segments (lateral and septal) on the apical four-chamber view one week after TAVI while the E/E' ratio did not change significantly.

**Table 2:** Standard echocardiographic parameters at baseline and one week after TAVI

	baseline	7 days post TAVI	*p-*value
Heart rate (beats/min)	74 ± 12	78 ± 14	.578

LVEF (%)	57 ± 8	59 ± 8	.116

LV end-diastolic volume (ml)	81 ± 29	88 ± 31	.063

LV end-systolic volume (ml)	36 ± 17	36 ± 18	.880

Maximum pressure gradient (mm Hg)	81 ± 30	19 ± 11	< .001

Mean pressure gradient (mm Hg)	47 ± 17	10 ± 6	< .001

Aortic valve area (cm^2^)	0.73 ± 0.27	1.86 ± 0.51	< .001

Aortic valve area index (cm^2^/m^2^)	0.41 ± 0.16	0.94 ± 0,44	< .001

Peak mitral E (m/s)	0.99 ± 0.35	1.26 ± 0.32	< .001

E deceleration time (ms)	236 ± 71	225 ± 80	.194

TDI-myocardial E' (cm/s)	6.8 ± 2.5	8.1 ± 2.1	.014

E/E'	15.6 ± 5.8	16.1 ± 5.1	.387

Data are expressed as mean ± SD. LVEF, left ventricular ejection fraction; LV, left ventricular; E, early transmitral velocity; E', early myocardial tissue velocity

### Left ventricular longitudinal strain and strain rate

For longitudinal speckle tracking strain and strain rate analysis out of a total of 1080 segments 1022 (94.6%) were accepted as yielding optimal tracking. The data of strain and strain rate analysis at baseline and follow up are shown in Tables 3 and 4. The global 2D longitudinal peak systolic strain in the apical long axis view, in apical four chamber, and in the two chamber view as well as average global peak systolic strain improved significantly from baseline to one week post TAVI (p = .009, p = .003, p = .004 and p < .001, respectively). This was reflected mainly in strain improvement in the basal and medial segments while strain in the apex did not change significantly (Table 4).

**Table 3:** Speckle tracking strain and strain rate data at baseline and one week after TAVI

	baseline	7 days post TAVI	*p-*value
Radial strain (%)	36.5 ± 13.7	39.7 ± 17.2	.458

Radial strain rate (s^-1^)	1.63 ± 0.44	1.97 ± 0.66	.004

Circumferential strain (%)	-18.1 ± 5.1	-18.9 ± 4.2	.607

Circumferential strain rate (s^-1^)	-1.40 ± 0.32	-1.59 ± 0.47	.031

Global longitudinal PSS (%)	-15.1 ± 3.0	-17.5 ± 2.4	< .001

Global longitudinal strain rate (s^-1^)	-1.01 ± 0.16	-1.14 ± 0.28	.005

Longitudinal PSS - APLAX (%)	-14.7 ± 3.6	-16.9 ± 3.7	.009

Longitudinal SR - APLAX (s^-1^)	-1.01 ± 0.23	-1.12 ± 0.34	.071

Longitudinal PSS - 4CH (%)	-15.4 ± 3.4	-17.2 ± 3.1	.003

Longitudinal SR - 4CH (s^-1^)	-1.00 ± 0.19	-1.15 ± 0.31	.007

Longitudinal PSS - 2CH (%)	-14.8 ± 3.5	-17.4 ± 3.8	.004

Longitudinal SR - 2CH (s^-1^)	-1.01 ± 0.23	-1.15 ± 0.28	.022

Data are expressed as mean ± SD. PSS, Peak systolic strain; APLAX, apical long axis view; 4CH, apical four chamber view; 2CH, apical two chamber view.

**Table 4:** Regional longitudinal speckle tracking strain data at baseline and one week after TAVI

	baseline	7 days post TAVI	*p-*value
PSS basal segments (%)	-11.6 ± 5.2	-15.1 ± 5.5	<.001

PSS medial segments (%)	-13.9 ± 5.2	-16.8 ± 5.6	<.001

PSS apical segments (%)	-19.2 ± 7.0	-20.0 ± 7.2	.481

PSS basal segments - APLAX (%)	-11.7 ± 5.9	-15.2 ± 5.3	<.001

PSS medial segments - APLAX (%)	-14.2 ± 5.6	-17.0 ± 6.1	.001

PSS apical segments - APLAX (%)	-17.6 ± 6.7	-18.5 ± 8.4	.937

PSS basal segments - 4CH (%)	-10.9 ± 4.2	-13.7 ± 5.0	<.001

PSS medial segments - 4CH (%)	-13.8 ± 4.4	-16.3 ± 4.7	<.001

PSS apical segments - 4CH (%)	-21.3 ± 6.9	-21.4 ± 6.2	.709

PSS basal segments - 2CH (%)	-12.3 ± 5.4	-16.3 ± 6.0	<.001

PSS medial segments - 2CH (%)	-13.6 ± 5.4	-17.0 ± 6.0	.003

PSS apical segments - 2CH (%)	-18.6 ± 7.1	-20.1 ± 6.7	.213

Data are expressed as mean ± SD. PSS, Peak systolic strain; APLAX, apical long axis view; 4CH, apical four chamber view; 2CH, apical two chamber view.

### Left ventricular radial and circumferential strain and strain rate

In the parastenal mid-ventricular short axis view 326 segments (90.5%) out of a total of 360 segments have shown a sufficient image quality for radial and circumferential strain analysis. The data of strain and strain rate analysis at baseline and follow up are shown in Table 3. Radial and circumferential strain remained unchanged one week after TAVI while radial und circumferential strain rate improved significantly.

### Intra- and Inter-observer variability

The inter- and intra-observer variability for longitudinal 2D Strain is 5.0 and 7.4% respectively. The inter- and intra-observer variability for radial 2D Strain is 10.3 and 12.8% respectively.

## Discussion

This study evaluated the changes of myocardial deformation using speckle-tracking strain imaging immediately after TAVI. We could demonstrate that patients with severe AS and preserved ejection fraction have impaired global longitudinal as well as circumferential and radial peak systolic strain and strain rate compared to previously published data of healthy individuals [17,25,26]. However, our patients are older than these controls therefore a comparison is limited since strain and strain rate are age dependent.

Our data also support the concept of "baso-apical gradient" of longitudinal contractility. At baseline, the patients with AS had reduced strain in basal and medial but normal strain values in the apical segments. This was already shown in the septal and lateral wall in patients with AS in a recently published study [27]. We could also show these differences in regional longitudinal deformation in the apical long axis and two-chamber view. In patients with severe AS increased pressure load and therefore LV hypertrophy, interstital fibrosis, and impaired subendocardial perfusion are present. These components are important factors that influenced LV regional and global longitudinal deformation.

Furthermore, in previous studies in markedly younger patients who underwent surgical aortic valve replacement an acute regional reverse remodelling after one week was documented [20,21]. In the present study with significantly older patients who were not able to undergo conventional surgical therapy an acute improvement in LV systolic function one week after TAVI was also detectable. This underlines the benefit of this new interventional therapy in this old co-morbid population.

We found a significant improvement of global longitudinal 2D strain, mainly of the basal and medial segments, while the strain values in the apical segments remained unchanged. The long axis function is partly dependent on the subendocardial fibres which orientation is predominantly longitudinal. These fibres are most susceptible to a reduction in coronary blood flow that occurs for example in hypertrophic hearts and left ventricular pressure overload [28,29]. Myocardial deformation imaging, in principal, is load dependent. The immediate excessive decrease in left ventricular afterload post TAVI itself but also a subsequent improvement of coronary blood flow in the subendocardial layer could explain the increase of longitudinal strain in the acute setting [29,30].

We did not find an improvement in radial and circumferential 2D strain in the short-term follow up of one week after TAVI. This observation underlines the more pronounced susceptibility of subendocardial longitudinal oriented fibres to acute changes in LV pressure load. Previous studies have shown an improvement also in circumferential and radial strain after surgical aortic valve replacement in the long term follow up [17]. The reverse remodelling after TAVI is possibly a stepwise process with acute longitudinal and at a later stage radial and circumferential improvement. However, Becker et al. documented significant changes in radial and circumferential strain and strain rate already 7 days after surgical AVR. But this observation was made in a highly selected patient group with isolated AS. The subgroup of patients with combined aortic valve disease did not show an improvement of circumferential and radial strain one week after AVR but after 6 month follow up. More than 50% of our patients had an aortic stenosis with combined mild to moderate insufficiency.

There are some general aspects when comparing our results to previously published data: The patient population in the presented study was older than the populations in all previous published studies. In our study, no patient was excluded due to significant co-morbidities that potentially influence LV deformation (arterial hypertension, mitral regurgitation and significant coronary heart disease). Despite relevant cardiac co-morbidities in this cohort of patients we could find a significant improvement of left ventricular longitudinal systolic function immediately after TAVI.

In summary, in addition to previously published studies with highly selective patient populations, we could show an acute improvement of LV function after TAVI in older and less selected patients, which represents more the "real life" clinical setting.

## Limitations

The present study includes only a small number of patients with no control group of matched patients with conventional surgical aortic valve replacement. However, patients with such a high co-morbidity will not undergo surgical valve replacement due to the high perioperative risk. We compared our baseline data with previous published data of normal individuals. Our patients were older than patients and controls in all previous published studies. Since left ventricular strain and strain rate are age dependent a comparison shows limitations. The long term follow up results are missing yet.

## Conclusions

There is an acute improvement of myocardial longitudinal systolic function of the basal and medial segments measured by 2D Strain analysis immediately after TAVI in old and multi-morbid patients with aortic valve stenosis. These data suggest that sensitive new echo methods can reliably detect early regional changes of myocardial function after TAVI before benefits in LVEF are detectable. In summary, in addition to previously published studies with selected patient populations, we could show an acute improvement of LV function after TAVI in less selected patients, which represents more the "real life" clinical setting.

## Abbreviations

AS: Aortic valve stenosis; AVR: (surgical) aortic valve replacement; GPSS: Global peak systolic strain; TAVI: Transcatheter aortic valve implantation.

## Competing interests

The authors declare that they have no competing interests.

## Authors' contributions

SS, GB and FK have designed and coordinated the study. SS and FK have analysed the data and written the manuscript. SS, GB, SS, KZ, AB, IP and WS have performed the echocardiography examinations for the study. GB, ML, VS and KS have performed the planning of the study, the patient selection and the TAVI interventions. All authors have read and approved the final manuscript.

**Figure 1:**
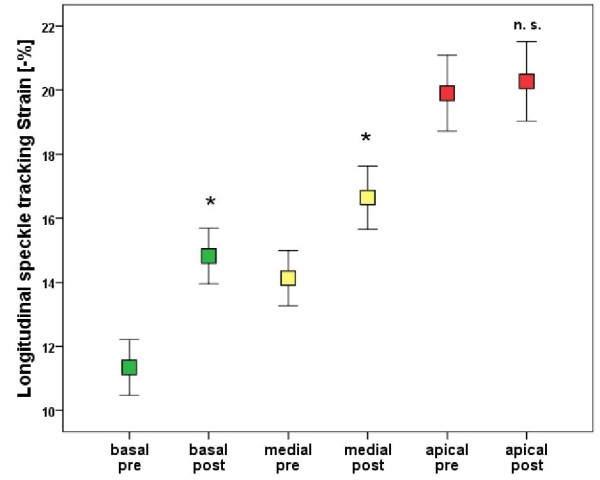
**Error bar analysis of regional longitudinal speckle tracking strain**.

**Figure 2:**
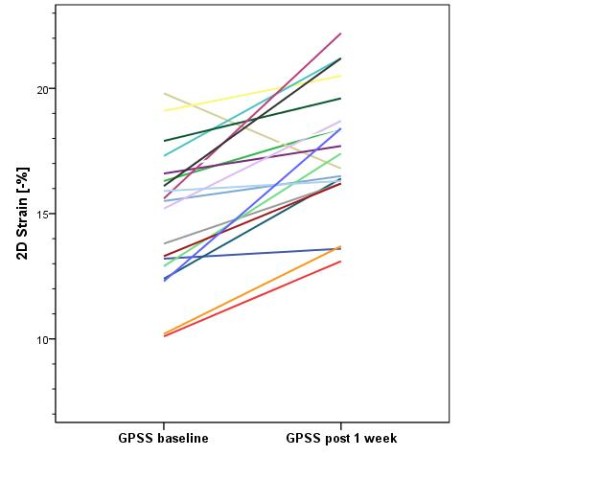
**Individual improvement of global peak systolic longitudinal Strain**. Note: The patient with acute impairment of longitudinal peak systolic strain developed atrial fibrillation after TAVI.

**Figure 3:**
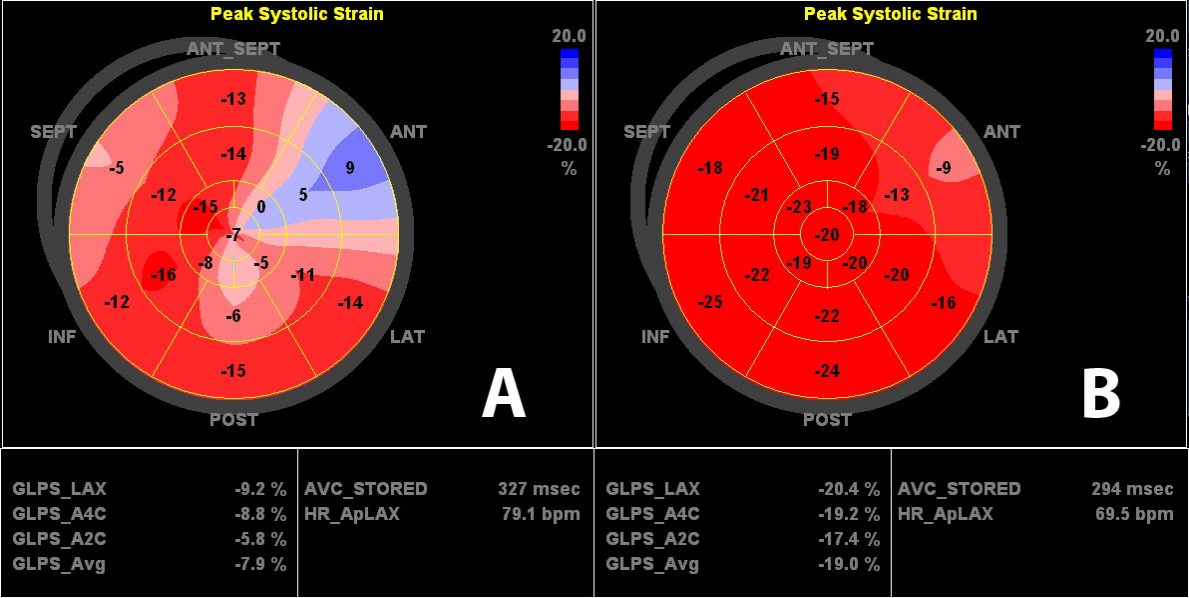
**Apical four chamber view longitudinal strain curves before (top) and one week post TAVI (bottom)**. There is an increase in longitudinal strain in all segments.

**Figure 4:**
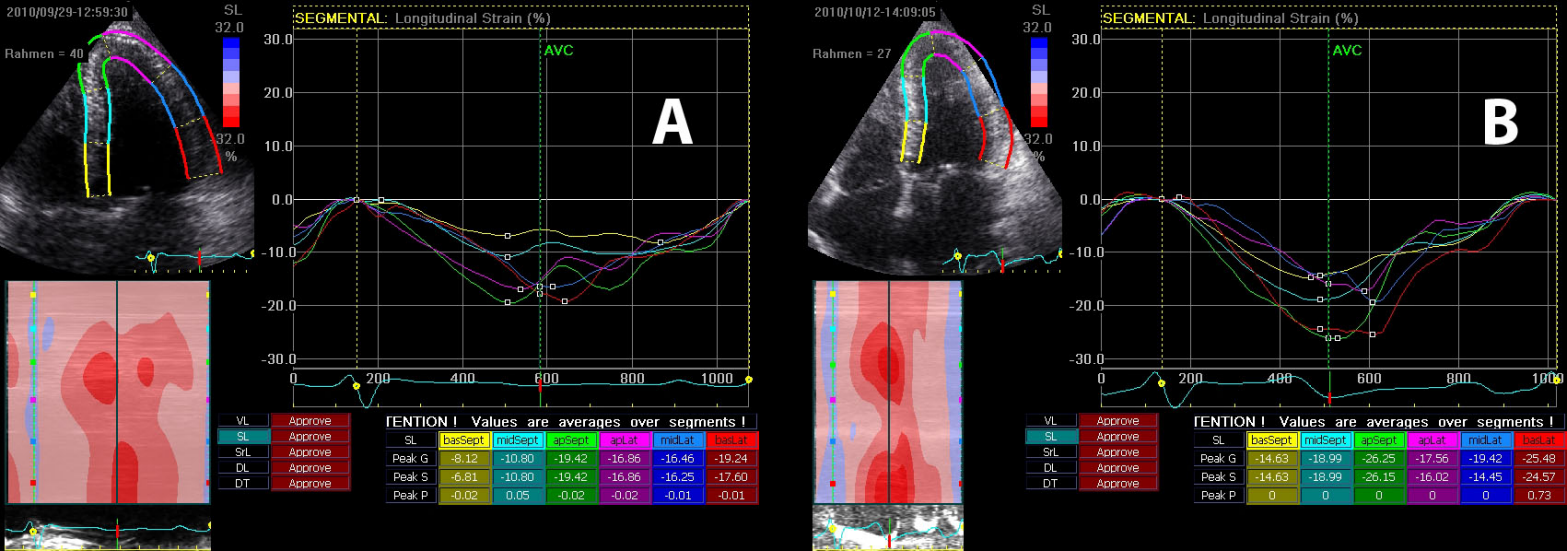
**Bulls eye diagram before (top) and one week post TAVI (bottom) with clearly visible improvement in longitudinal strain**.
